# Reconstructing Dengue Exposure Dynamics and Assessing the Public Health Impact of Vaccination Scenarios Using Mathematical Models in Brazil

**DOI:** 10.64898/2026.10.15.26365586

**Published:** 2026-10-16

**Authors:** Youngsuk Ko, Cleber D. Santos, Angkana T. Huang, Henrik Salje, Derek A.T. Cummings, Albert I. Ko

## Abstract

Brazil experienced its worst dengue epidemic in 2024, with an increase in severe outcomes among elderly individuals, highlighting the need for vaccination strategies informed by population immunity. We reconstructed state- and age-specific immune profiles by fitting an age-structured catalytic model to dengue surveillance data from 2000–2025. This inferred immune profile was incorporated into an SIR-type transmission model to project disease burden and to evaluate the public health impact of vaccine allocation strategies. We observed substantial spatial heterogeneity in accumulated immunity, which was closely associated with projected burden and optimal allocation. Strategies prioritizing states with lower historical incidence and larger susceptible populations yielded greater benefits. Under limited vaccine supply, targeting children resulted in the greatest reductions in DALYs, whereas targeting elderly populations achieved smaller DALY reductions but larger reductions in mortality. We further showed that accelerated vaccine supply markedly increased impact, while allocation choices remained critical when availability was constrained.

